# Implementation and Evaluation of an Integrated Care Team for Refugee Health: A Case Study in Ontario, Canada

**DOI:** 10.5334/ijic.9815

**Published:** 2026-07-08

**Authors:** Catherine E. Tong, Alexandra Whate, Wajma Attayi, Debbie Engel, Paul Stolee

**Affiliations:** 1 School of Public Health Sciences, University of Waterloo, Canada; 2 Community Healthcaring Kitchener-Waterloo, Canada; 3 Camino Wellbeing & Mental Health, Canada

**Keywords:** refugees, primary care, care transitions, integrated care

## Abstract

**Background:**

Refugees face significant barriers in accessing primary care, often resulting in poorer health outcomes. This case study evaluates the implementation of a multidisciplinary Integrated Care Team (ICT) for refugees, in Ontario, Canada. The aim of the ICT was to transition refugees into primary care practices.

**Methods:**

A mixed-methods process evaluation was conducted to assess the implementation and outcomes of the ICT program. Data sources included ethnographic observations, in-depth interviews with ICT staff, clinicians, and refugee patients. Thematic analysis was applied to qualitative data, and program database metrics were analyzed to evaluate patient transitions and service use.

**Findings:**

In its first year, the ICT transitioned 664 refugees to primary care, surpassing its goal of 300. Key successes included improved collaboration, enhanced system navigation, and reduced pressure on refugee clinics. Refugee participants reported feeling supported through the ICT program, emphasizing the importance of personalized care and assistance in navigating the healthcare system. The program improved access to primary care, facilitated timely referrals, and addressed social determinants of health.

**Discussion:**

The ICT program demonstrates the potential of integrated care to enhance refugee health outcomes. Flexibility, collaboration, and proactive support were essential to its success. Future research should explore long-term impacts and scalability.

## Introduction

There are currently more than 43.4 million refugees worldwide [1] with approximately 75% hosted in low- and middle-income countries and 25% in high-income countries [2]. Refugees are at a higher risk for poor health outcomes [3], and providing accessible, high-quality health care for refugees presents unique challenges for health systems and individual providers [4]. Even if they resettle in high-income countries with universal health care, refugees often face barriers to care, including language differences, unfamiliarity with local health systems, and complex acute and chronic health needs that stem from their pre-migration experiences [5,6]. Refugee patients are also more likely to present with complex psychosocial needs, past traumas [7], and, for some, mistrust of health care providers [8,9]. In addition, refugee patients often face settlement challenges; depending on the supports available in the receiving country, many social determinants of health (e.g., housing, employment, social support) remain unmet. Although the standard of living of refugees who have resettled in higher-income countries – particularly countries with strong social welfare systems –may improve, many refugees continue to experience unmet housing needs, un-/under-employment, food insecurity, psychosocial challenges (e.g., separation from family, potentially limited social networks, etc.) and discrimination [3,10].

In Canada, where our work is situated, primary care is the entry and access point to the broader health care system [11] and is intended to be the locus of care coordination [12]. For refugees, primary care also plays a role in connecting patients to the wider basket of social supports available. Refugees in high-income countries, however, tend to underuse primary care services and overuse emergency room services [3]; this limits the patient’s ability to benefit from the continuity of care, interprofessional care, and system navigation support that primary care could provide.

We define integrated care as “an approach to overcome care fragmentations, especially where this is leading to an adverse impact on people’s care experiences and care outcomes” [13, p 2]. In addition, integrated care models typically aim to connect care provided across different and disparate part of the health care system and often take a patient-centered and/or team-based approach. Knowing that refugees in high-income countries tend to overuse emergency services and underuse primary care, integrated care models have the potential to greatly improve their care experiences and care outcomes and may also reduce strain on emergency services. In a global review of 55 initiatives aimed to improve primary care for refugees [14], interventions have typically focused on: 1) developing the skills of refugees (e.g., awareness, learning how to navigate their new health care system); 2) enhancing the skills of primary care staff (e.g., training in cultural safety and trauma-informed care); 3) introducing various types of communication and interpretation supports; and 4) system and/or service integration models (e.g., linking refugees to interdisciplinary health care teams and primary care “medical homes”).

In Canada, designated refugee health clinics have emerged as a model to provide initial care to individuals upon arrival, however timely integration into the broader primary care system has been, and remains, a challenge [15,16]. Today, refugee clinics in Canada are serving more patients than ever, with patients receiving a higher volume of monthly appointments and clinicians working ever-increasing hours to keep up with the growing demand [17]. Under the Interim Federal Health Program, these clinics serve privately sponsored and government assisted refugees (GARs), and asylum claimants whose status is pending. While Canada has long accepted refugees, 2023 was a record-setting year, with more than 144,000 asylum claims received (a 1.5-fold increase from the year prior). Nearly half of these claims were made in the province of Ontario, where our case study is situated. There is a need for more evidence on effective models and practices in caring for refugees, including models that are functioning within the current context of a health care system in “crisis” [18] and incorporating record-setting numbers of refugee claimants. Understanding the logistical and financial challenges of implementing integrated care for this medically complex population, and finding solutions for practical implementation, is needed for health systems seeking to improve integrated care [19]. Failure to do so is resulting in exemplary but overstretched refugee health clinics that are unable to absorb new patients, and vulnerable patients are not receiving care [16].

## Program Description

### Setting and Context

In 2019, the Ontario government introduced its largest and most recent initiative to attempt to integrate care, with the formation of 24 “Ontario Health Teams” (OHTs). These OHTs are meant to advance care integration with system navigation supports and care coordination [20]. In October 2020, the Kitchener-Waterloo-Wellesley-Wilmot-Woolwich (KW4) region of Ontario formed an Ontario Health Team (OHT) focused on priority populations, including refugees. The region is a designated resettlement area for refugees, including those that are GARs, privately sponsored, and asylum claimants. Two local specialized refugee health clinics provide initial primary care to refugees during their first year in Canada, however both clinics were facing significant capacity constraints and were having difficulty transitioning patients out of the refugee-specific clinic and into the regular primary care system. This resulted in significant backlog and an inability for the clinics to take on new refugee patients that continued to arrive in the region. To address this, community partners came together, with financial support from the KW4 OHT, to develop and implement an Integrated Care Team (ICT) program for refugee patients, with the aim to help transition patients to the broader primary care system in a supportive manner. This case study examines the implementation process, outcomes, and lessons learned from the first year of the ICT program.

The Refugee Health ICT program launched in January 2022. Both local refugee health clinics had extensive waitlists (approximately 1000–1300 at each clinic) of refugees applying to become rostered patients, and although the clinics were only intended to be used for up to one-year post-arrival, both clinics found that they were unable to transition patients to regular primary care clinics in the region. In the years leading up the ICT, each clinic was transitioning no more than 5% of patients each year, sometimes far fewer. While on the waitlist, sometimes for years, refugee patients use local walk-in clinics and emergency rooms to access care, but this care lacks continuity and those providers are often not specialized or equipped to meet the needs (e.g. mental health concerns, tropical diseases) of this patient population. The ICT program was designed to alleviate pressure on the refugee clinics, by providing a range of supports to help transition medically stable refugee patients to other primary care providers (PCPs) in the region. The team provided supports to the refugee patients who were moving to other providers and to the receiving PCPs.

### Program Structure and Objectives

The ICT brought together an interdisciplinary team of staff from multiple organizations, including: a) three Newcomer System Navigators from a community services agency focused on providing social and mental health services to newcomers; b) a Community Pharmacist and a Case Manager/ICT Coordinator from one refugee clinic; c) a Discharge and Intake Coordinator from the other refugee clinic; d) a Care Coordinator from regional Home and Community Care Support Services (HCCSS), a publicly-funded agency that provides in-home nursing, physiotherapy, occupational therapy and personal support workers who assist with activities of daily living; and, e) a Case Manager from a refugee resettlement agency that provides health and wellbeing support, housing supports, and training and employment services. Leadership for the team was provided by Directors (non-clinician administrative leaders) from one refugee health clinic and the community services agency.

The program provided two levels of support: **Level 1** support included basic transition support from the Discharge Coordinator that included appointment booking and medical record transfer to the patient’s new PCP. **Level 2** support included more intensive support including follow-up and connection to additional ICT services. The objectives of the ICT program were:

1. To successfully transition 300 medically stable refugees, who have been in Canada for more than a year, to non-team-based PCPs within one year.
2. To provide refugees with easier access to community resources and support services.
3. To support refugees to become more independent in navigating the health and social system to access the supports they need.
4. To increase the knowledge of refugee patients such that they know with whom to connect and where to go when they need help.
5. To support PCPs to enable them to take on more refugees as patients by having them access a team of inter-disciplinary professionals and interpretation services to better provide care.

## Methods

We employed a mixed-method process evaluation approach [21] to monitor and document the ICT program’s implementation, and to understand the relationship between specific program elements and program outcomes. Our evaluation team included the ICT program leadership team and three university-based researchers [CT, AW & PS] with expertise the evaluation of health care initiatives and collecting data with immigrants and refugees in multiple languages.

The overall aims of this evaluation were:

1. To track and describe the implementation and evolution of the ICT program and to describe the activities of the ICT.
2. To identify barriers and facilitators to implementation of ICT activities.
3. To determine the characteristics needed for PCP clinics, staff, and patients to implement successful ICT programs.
4. To understand the types of complexities experienced by patients who have been served by the ICT program and what their experience has been.

### Data Sources & Analysis

**Figure 1:**
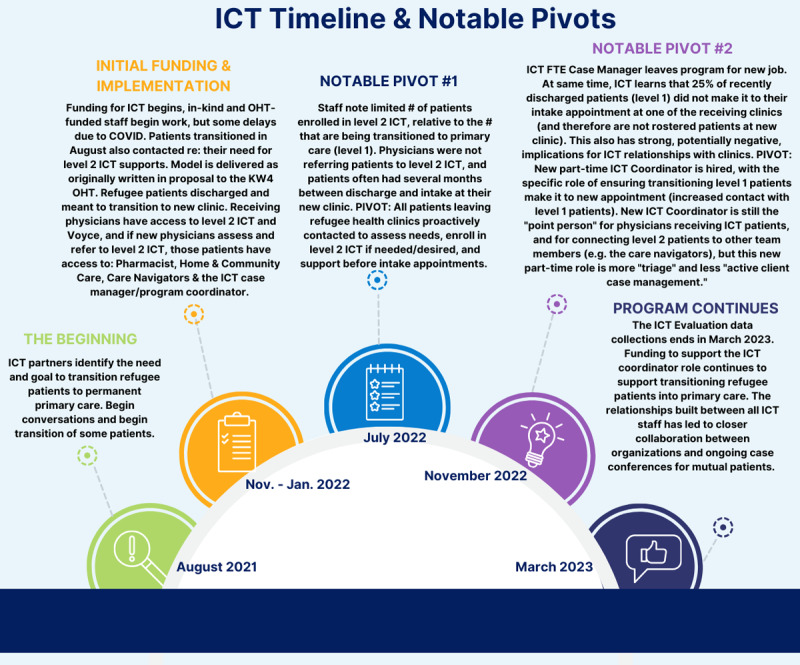
ICT Timeline and Notable Pivots.

Our data sources include ethnographic field notes, semi-structured interview transcripts, and program statistics (e.g., # of patients served, # of patients transitioned, # of appointments attended, etc.) collected by the ICT team. The university-based evaluation team attended and took ethnographic style notes at 32 ICT team meetings, between May 2022 and March 2023. The notes documented ICT discussions, team decision making processes, and noted barriers and facilitators to implementing the new initiative. The evaluation team completed in-depth, semi-structured interviews with eleven ICT staff members, two primary care clinicians receiving ICT patients, and eight ICT refugee patients. The ICT team supported participant recruitment by sharing study materials (e.g., a study information sheet and email), and for ICT patients, providing a verbal description of the evaluation project. The ICT frontline staff who were interacting with patients were asked to present the study information sheet to all program participants that they interacted with in the Fall of 2023 (September to December); these frontline staff generally spoke the same languages as their assigned patients. Patients who were interested in participating consented to have their name and contact information passed to the research team, along with their preferred language of communication. The research team members then contacted ICT patients, using Voyce (a remote medical interpretation platform) interpretation services if needed. The ICT program supports people who speak dozens of languages. Voyce offers interpretation in 240+ languages and participants could select interpretation in any language of their choosing. Five participants requested interpretation for their interviews, in Arabic (n = 5), Somali (n = 1) and Tigrinya (n = 1). The remainder were completed in English Interviews were digitally recorded, cleaned and anonymized by a trained research assistant. Meeting notes and interviews were all uploaded to NVivo, a qualitative software analysis program, in which the team completed a thematic analysis [22]. Our thematic analysis process included: 1) familiarization with the data (all team members reading 2 transcripts from each participant group), 2) development of a codebook with definitions, 3) reviewing the codebook with the ICT team, 4) two coders line-by-line coding all transcripts in NVivo, and 5) analytic memoing and meetings throughout, both between the two coders and with the wider team. With a focus on documenting program implementation, all changes to team processes were extracted and a timeline of program changes was created to capture the evolution of the ICT program (See Figure 1).

The ICT also provided the research team with aggregate data from their program database (e.g., number of patients served, number of patients transitioned to PCPs).

### Involvement of persons with lived experience

Refugee patients from the ICT program participated in interviews, however given their limited English and other intersecting factors (e.g., they were very much focused on survival and re-settling their families), we were not able to fully include them in all aspects of the research process; however, those working directly with refugee patients on a daily basis were included in every aspect of the research process, from conceptualization through to analysis and reviewing and providing feedback on final reporting. In addition, several members of the ICT team were former refugee patients themselves, bringing that lived experience to each phase of the evaluation (e.g. they reviewed our interview guides and provided feedback on the types of questions and phrasing, participated in monthly research meetings, and reviewed participant summary reports for language and accessibility prior to distribution).

### Ethics

Our study received ethics clearance from the University of Waterloo’s Office of Research Ethics (ORE # 44477).

## Findings

Aligned with the aims of this work, our findings are related to both the implementation of the ICT program, and its impact. We discuss the impact on the team, and on the individual patients that we interviewed. The timeline in Figure 1 provides an outline of the program, and two important pivots made during implementation.

### Program Implementation: Success, lessons learned, and persistent barriers to implementation

Between September 2021 and March 2023, the ICT program transitioned 664 patients from two refugee health clinics to PCPs. Of these patients, 621 received level 1 support, that is, they were provided with assistance in booking their first intake appointment and medical records were transferred to their new clinic; 43 of these patients (24 families) required **Level 2** support from the ICT. This exceeded the initial goal of transitioning 300 patients, demonstrating the program’s efficiency and the high demand for its services. ICT staff spent a total of 565 hours serving **Level 2** patients (data related to time spent serving **Level 1** patients was not available for this evaluation).

#### Implementation: Discharge & intake processes

The Discharge and Intake Coordinators used specific indicators to signal that a patient is ready for discharge from the refugee clinics to regular PCPs. These indicators were:

1. The patient is deemed medically stable by the health care team without any major complexities or issues; and/or complexities are well-managed;
2. The patient can book an appointment relatively independently (may include an assessment of language skills or ability to access interpretation);
3. The patient has a demonstrated ability to attend appointments (e.g. patient shows up to current appointments and has access to transportation); and
4. There is an appropriate doctor willing to take on the patient (may include language and cultural considerations). Finding a ‘good match’ between patient and physician is important for success.

Patients who do not meet these criteria stay as rostered patients with the refugee clinics for as long as the coordinators and physicians deem necessary, and in some cases this takes years. The intake process varied depending on the new PCP clinic to which the patient was being transitioned. Staff reported that different clinics have different protocols and intake procedures, including differences in the ways that medical records are transferred and the ways in which clinics operate (i.e., refugee clinics often operate in a much more flexible manner, able to accommodate late arrivals, numerous family members arriving at an appointment made for one, etc.). There were also differences in the follow-up processes (e.g., some clinics accept email appointment requests), and language capabilities of staff (i.e., while clinics are legally required to provide interpretation services for communication between physicians and patients, there is no such requirement for administrative staff and others who communicate with the patients). Staff noted quite a few issues that arose during this process, including files not being transferred successfully, patients missing intake appointments, patients using intake appointments for medical issues (when it is meant to be a brief “meet and greet”), and patients missing out on needed services between discharge from the refugee clinic and intake at the new PCP. This latter issue was the precipitating factor for **notable pivot #1** (Figure 1) in the way that the ICT program was implemented. At the start of the program, patients were discharged from the refugee clinics and it was up to the new PCP to request ICT supports after intake at the new PCP had occurred. Due to the issues identified, the Discharge Coordinator began referring all outgoing refugee patients to the ICT coordinator for assessment immediately upon discharge to ensure that those needing ICT supports were identified prior to their intake appointment with the new PCP.

#### Implementation: Team composition & roles

Establishing the roles on the ICT took some time and planning and ensuring that ICT team members understood each others’ roles and boundaries was important in the development of the program:

*“As we onboard more team members […] we started more team meetings to know each other, define our roles, that was a big piece of it, make sure that we all knew kind of the boundaries of each other’s roles, but also what each other’s roles could offer, seeing those not everyone had worked in kind of this capacity beforeWe also made sure that each team member had a say so that nothing that we planned was set in stone because we were very understanding of the fact that the program and our kind of criteria for it was going to have to be adaptable and flexible.”*

Notably, many staff members mentioned the importance of flexibility in the roles of the team members, as several roles were working beyond the usual scope of their profession. Having ICT members with a variety of backgrounds was seen as a strength of the program as it allowed patients to receive more holistic care. The multidisciplinarity of the ICT team and the sharing of expertise, skills and experience was observed to be a critical component of the successful implementation of the program.

#### Implementation: Team building & new collaborations

In observing the ICT meetings, we noted increased comfort and collaboration between ICT staff members over time. For many members of the ICT, this was their first time meeting and working with staff from the other organizations. One staff member remarked:

*“Up until ICT happened [the refugee health] clinics weren’t connecting. And they weren’t connecting with mental health services or those community supports. They were very insular. I would say the biggest impact is the bringing together of home care, and the medical models and our organization and [local refugee settlement agency] to really clearly understand how our work can work together to help support.”*

The collaboration between the organizations involved was seen by staff as one of the most important aspects of this work, as they were able to provide more holistic and coordinated care.

#### Persistent Challenges: Complex work in a short timeframe

Many staff noted that the work involved in successfully transitioning and caring for this patient population is much more complex, and more time consuming, than average, noting specifically that the numbers in the program may not be a true reflection of the amount of effort that has happened, nor of the true impact of the work:

“*It’s not only the number of impacts being made, but the quality of those impacts being made. Because you can make like 100 contacts [of patients to new PCPs], but you can transition them and they could be in a really precarious situation, with no help, right. Or you can have, like 40 patients and be able to like really spend more time listening to their stories and creating sustainable care plans […] I love the opportunity to work with people more so that we’re not putting out fires, but we’re preventing them from relapsing into the program again.”*

All ICT staff stated that some refugee patients, particularly those in the ICT program, have complex needs that are often intersecting and all-encompassing. As one staff member noted, we can’t “*talk to them about mental health needs while they don’t have food, and they don’t have secured housing*.” As such, providing more comprehensive health and social supports was critical for the wellbeing of the patients and the ability to move these patients to a longer-term health care provider. Several team members noted that one year is not enough time to “hit their stride.” One team member who had previously worked on a similar integrated care team felt that her prior team took several years to gel as a team, firm up their model, and start to demonstrate strong impact. The initial funding for the ICT was allocated for a period of 18 months.

#### Persistent challenges: Physician recruitment and program uptake

ICT members repeatedly discussed the importance of physician involvement in the ICT. It is fundamentally impossible to move patients out of refugee clinics and into a more permanent medical home, without practices *willing and able* to take them on.

*“The number one issue is a lack of family doctors, or practitioners just generally being able to attach folks…So, the lack of doctors just means that you sort of end up having really no need to take on more patients… If you have a handful of doctors who are willing to take on one family a year, and then a few clinics who can take on the bulk of that, that’s not a system that’s going to be sustainable in the long run.”*

Several ICT members have worked to establish relationships with physicians, to facilitate the transition of patients. These can be complex relationships that need to be nurtured. ICT had a limited number of instances in which refugee patients and new physicians did not interact well, and the ICT tracked and addressed issues as they arose. For example, at one point approximately 25% of patients who were meant to transition to a new clinic were “no shows” and missed their initial appointment with the physician meant to be their new doctor. **Notable pivot # 2** was introduced in response to this: the ICT introduced a part time staff member who was specifically tasked with supporting appointment setting and reminders, as ICT recognized that this pattern of “no shows” could compromise the relationship with the clinic, and their willingness to receive refugee patients in the future.

The ICT model was meant as “the carrot” to entice and support physicians who were willing to take on new refugee patients.

*“…that’s the whole point of this team, to eliminate that gap. Because when we transition from our clinic to a new doc, they’re focusing on the medical needs of the patient, whereas this team is bridging that gap to fill in the social determinants of health…it makes it easier for the doctors and the patients themselves. That warm connection.”*

After adjustments were made to the program, the ICT staff felt more confident that patients experienced more continuity in their health care, since ICT staff were involved earlier on in their transition.

#### Persistent challenges: Working a resource-scarce health and social care system

Many ICT members discussed the emotional toll of working in a broader health and social care system that is not sufficiently supporting refugees. For example:

*You have to learn boundaries, right? You have to learn okay, this, is this my job Because if you try to take on everything, you’re gonna burn out, right? Especially when it comes to really tough things such as housing, like that is a situation where often you have to say to patients, this is the best option we have and it’s still not ideal, right?*

An additional complication, shared by both ICT leadership and participating clinic staff, was that refugee clinics provide a very comprehensive and flexible model of health care, in which entire families can attend an appointment, several issues can be discussed in one appointment, and arriving late or missing appointments is generally accommodated. While this level of flexibility and compassion is warranted for newly arrived refugees, it becomes difficult for refugee patients to move to new clinics, which are not nearly as flexible. As refugee patients become more aware of the shortcoming of the broader health care system for the general public, there is little incentive to leave for a new practice. One clinician explained:

*“That’s the other thing that I would love…education around the different parts of the Ontario health care system…some education around, you know, family doctors want everyone who’s going to be seen to have an appointment, don’t bring all four children in a 10 minute spot… that’s where the exhaustion comes in. Right? And that’s really hard to educate around. Because of the refugee clinic, they do it like that and probably book accordingly. I don’t do that.”*

Another clinician similarly noted:

“*The first 30 days, by all means, I will see you every day, if that’s what it takes, right… but, hey, as you get more and more stable, we will start to make you more independent. And then that – if that mimics what, you know, a physician in our community can do, then that transition, it just becomes less painful… if your end goal is to get patients to move out of your clinic, what’s the incentive of somebody getting great service to leave your system?”*

### Understanding ICT patients’ experiences with the broader health care system, and the ICT program

#### Experiences with the health care system

The ICT patients shared numerous barriers to care that they had experienced, including wait times and transportation challenges. They also shared key facilitators, chiefly having a provider or clinic staff who speak languages that they are comfortable speaking. Language, communication and interpretation functioned as both a barrier and facilitator to care, depending on the context, patient and provider.

#### Barriers to accessing care

Several participants indicated that despite having a new family doctor and a referral made, they had trouble accessing the specialist care they needed in a timely manner. For one participant, the wait time for a specialist was made even longer due to lack of interpretation services. It is not uncommon to wait for long periods of time for specialist services in Ontario; we learned that refugee patients may wait even longer, if interpretation services are not available when needed. Interpretation assistance, or lack thereof, was the more persistent and commonly cited barrier to accessing care. One participant described the frustration that she has experienced:

*“Every time we go to the doctors, they would tell us that the clinic called and, yes, they called, but we need someone who speaks in Arabic to call us and tell us what is required, because we know that when they’re speaking, they’re saying one of our names, but we don’t know what they’re saying […] The doctor is, he speaks Arabic, but none of the staff speaks in Arabic.”* -Female, age 46

A few participants noted that transportation is a barrier to them accessing health services. One participant noted the difference between their ability to access their initial refugee health clinic, and the transportation issues they face when trying to access their new family doctor:

*“Previously our doctor was near our home near downtown, so it was not too far, it’s like 15 to 20 minutes, so it was easy for us to go to our doctor. But now, she’s too far, even, we cannot travel by bus. It’s very far we cannot reach after one hour.”* Male, age 32.

Location of the clinic and access to transportation should be considered when patients are transitioned to their new family doctor.

#### Facilitators to accessing care

Participants described several factors which made accessing health care easier. Several participants noted that the fact that their physician spoke their language was of great benefit:

*“…the physician speaks our language…we were able to explain to him any new pain that comes up, any new symptom, and any modification in the medication or change in the dosage. We were able to understand him when he talks, when he talked about that. So, the treatment we received were very excellent.”*-Male, age 75

In addition to speaking the same language, participants who were rostered at clinics that provided walk-in appointments found accessing care easier than it was at clinics that required appointments. Providing care in the language of the patient, and providing flexible, on-demand appointment times were both facilitators to accessing care.

#### Experiences with the ICT program

Participants were overwhelmingly positive when describing their interactions with ICT staff and the services provided. Participants repeatedly expressed feelings of relief and gratitude, and discussed a wide variety of supports provided by the team, including referrals, medication management and facilitating connections to the broader health and social care systems.

Several participants indicated that the ICT gave them feelings of relief, and felt that their lives were made easier by their connection with the ICT:

*“…this experience has been really beneficial and really mind easing. It makes everything easier for the newcomers in general, it really helps through the services and the facilitations provided”- Male, age 35*

Some participants described that their situation prior to their connection with the ICT team was quite dire, with one participant explaining:

*“Our situation was really bad when we met [name of ICT staff member] and her team and – I cannot even imagine how we would have survived without their help and their services or their systems. So, we did not even have anything to eat or anything to support ourselves. So, I think we really needed their help and without them we could not have survived.”* -Female, age 48

Not a single participant reported any negative experiences or outcomes due to their interactions with the ICT, and several asked the interviewers to pass on their thanks to specific ICT members, noting how the team had treated them with kindness and compassion.

#### Services provided by ICT

Participants described a wide variety of services and help provided by ICT members. Medical supports were largely related to system navigation and referrals, and included: helping them access physiotherapy, psychiatry, and optometry services; facilitating timely referrals to mental health supports; registering for programs that provide financial assistance for assistive devices; medication reviews and reconciliation; and making and getting to appointments. Non-medical supports included referrals to housing, food and other community services.

Several participants were provided with connections to psychological services through the ICT, with one participant sharing that the ICT was able to connect her daughter with a counsellor when her daughter was experiencing life threatening mental health issues: “*They [the ICT members] know my daughter is suicidal. They got [daughter] like, a counselor…I feel better now that [my daughter] has somebody like a counselor*.”

Over half of participants indicated that the ICT helped them with their medications, including providing medication consultations in home. One participant described how the ICT pharmacist was able to communicate with their physician to straighten out some mix ups with medications:

*“They also helped me with my medications. I had a problem pertaining one of the medications wasn’t possibly taking correct, correctly. And there was a mix up of medications, so [the ICT pharmacist] helped me […] They also organized the process of how the medications are going and also getting information with the doctor”* -Male, age 45

Many participants reported that the ICT helped them to book, and keep, appointments with their new family doctor, some describing that the ICT member is the person they call to book the appointment on their behalf. One participant explained that before they were in contact with the ICT, they missed multiple appointments with their new family doctor, and that the ICT was now intervening to help them reschedule their appointments.

Many participants described the ways in which the ICT was able to connect them to services in the region, including such things as passports and identification services, by helping with paperwork and applications, and by connecting patients to the YMCA. Participants also reported that the ICT was able to help them connect with housing services. Additionally, several participants reported that the ICT helped them to access programs for their children, with one participant even noting that an ICT member provided a bicycle for his son:

*“They also helped with activities for the kids. So, they did everything…Even one time I told them that my son wants, wanted a bicycle. They tried to get him one. So, I want to thank them very much. They’re a good team.”* Male, age 37

The services and connections provided by the ICT went above and beyond medical services and included a range of social services that improved participants’ quality of life.

## Discussion

This case study describes both the impact and implementation process of an integrated care model for refugee health in its first year. During its development and evaluation period, the ICT successfully transitioned over 600 patients to new PCPs while providing varying levels of support, exceeding its initial goal. Key lessons learned during this period of implementation are summarized in Table 1. The need for two significant pivots in the program’s first year highlights the importance of flexibility in implementing new care models. Our work demonstrates that a new refugee health integrated care team can have substantial impact in its first year but several pivots and modifications to the model or processes may be required. This is consistent with the implementation of many integrated care initiatives, which often require pivots, adaptions and flexibility in order to be successful [23,24].

**Table 1:** Lessons learned in the implementation of an integrated, team-based care program for refugee patients.


Flexibility and adaptability are crucial when implementing an integrated care model for refugees. The program had to evolve and adjust its processes based on identified issues and patient needs. Proper preparation of patients for transition from specialized refugee clinics to regular primary care providers (PCPs) is essential. Patients need education on the differences in care models to manage expectations. Multidisciplinary teams with diverse backgrounds and expertise contribute to more holistic patient care. The program’s success relies heavily on building and maintaining relationships with PCPs willing to accept refugee patients. Where PCP shortages exist, this is critical. The impact of integrated care programs may not be fully captured by quantitative metrics alone, or within a short time frame. The complex nature of caring for refugee populations should be considered when evaluating these programs.


At the time of writing this manuscript, we are pleased to confirm that funding for the ICT program has been extended into 2025, well beyond the initially planned 18 months, and has now transitioned more than 2500 patients. More than a decade ago, Canadian researchers called for an increase in integrated community-based primary care interventions for refugees, and interventions that include meaningful connections to the broader social support network and systems (e.g. with system navigators) [25]. This program directly, and successfully, addressed those calls to action. ICT team members noted the value in bringing together several disparate pieces of the health and social care system(s) who were previously not connecting. This bridging included bringing together, through the ICT program, two refugee clinics, a settlement agency, primary care, numerous specialists, home and community care, mental health services, and programs providing support for food, housing, and accessing medical equipment. Canadian refugee clinics are globally viewed as exemplary providers of integrated, patient-oriented, interdisciplinary care [16,17]. This exemplary model, however, comes at a cost: clinicians working in Canadian refugee clinics are working excess hours, experiencing burnout, vicarious trauma and financial strain [17]. The cost to patients includes not receiving any care or turning to services such as emergency rooms and walk-in clinics, which lack consistency are not suitable for ongoing primary care [16]. The ICT program helped to defray these costs to refugee health clinics and patients, by connecting medically stable patients to a much larger health and social care system of disparate but valuable supports.

Bridging to primary care and ensuring complex and high-needs patients were supported to make the transition, was the most labour-intensive aspect of the program, and the piece of the program that faced the most barriers. With the way that primary health care is set-up and funded in Canada at present, there is little incentive for providers to take on the most complex patients, who will require a higher degree of care coordination [26]; this is a function of the system, and not necessarily the willingness of individual providers. Without primary care practitioners and teams willing and able to take on refugee patients, this program would not have been successful, and refugee patients would have likely continued to access the health and social care system in ineffective ways (e.g., in crisis, at the emergency room), or not at all. Participants also emphasized the importance of expectation setting and once settled, educating refugee patients about the realities and limitations of the regular (non-refugee) health care system. Just like their Canadian counterparts, refugee patients will be expected to accept short appointments that deal with one issue at a time, wait times, health care information that doesn’t travel well between providers, and a system that is better at being reactive than preventive [27]. We confirmed through this work that refugee patients are experiencing the ill-effects of a health care system that still lacks integration.

We also confirmed through this research that refugee patients are a highly heterogenous group, with diverse needs, skills and abilities. We interviewed patients who received the more intensive Level 2 supports, but there were also hundreds of Level 1 patients who were tech savvy, navigating our complex systems, proficient or becoming proficient in English, and who made the transition to their new primary care practice with relative ease. Parsing out the groups based on their degree of need, rather than offering more intensive supports to all refugee patients was a more resource effective way to get the wrap-around, team-based care to those who needed it most. Similar to the Newcomer Clinic Model described by Kohler and colleagues [16], we too found that it was critical to assess the readiness of refugee patients who may be able to transition to a new practice, and then proactively support the transition. ICT transition supports included a readiness assessment, document transfers and staff with time allocated to support the transition in a very hands-on manner (e.g., making appointments on the patient’s behalf, accompanying them to initial appointments).

Finally, our work suggests that integrated, preventive and team-based care may have resulted in some cost reductions; the ICT team, for example, helped prevent missed appointments, provided proactive and critical medication reconciliation and guidance, transitioned patients into PCPs that were less resource intensive to run than a typical refugee clinic, and worked “upstream” [28] to ensure basic needs such as housing and access to food were met. Patients shared examples in which the team connected them and their families to potentially lifesaving supports and services. At the same time, we recognize that these sorts of teams are expensive, and it is highly unusual in the current Canadian health care system to see clinical team members accompanying patients to appointments or making calls on their behalf. Truly integrated care programs are known to be complex, and require resources and activities (e.g., care coordination, communication across sectors) that are often not recognized or adequately renumerated by existing payment schemes [29]. Investments in people and care, however, are not and should not solely be informed by cost-savings, and this was a value that was clear in our interviews with the ICT staff. Whether Canadian-born or a refugee, some patients will require intensive supports for an extended period, and we may decide to invest more because it is the right thing to do and reflects the values upon which our universal health care system was created [30].

### Limitations

This case study has several limitations. Firstly, this study examines a single regional program, which may limit generalizability to other contexts. In addition, the evaluation covers only the first year and a half of implementation, which will not capture long-term outcomes or sustainability issues. Finally, this evaluation focused primarily on process measures and qualitative experiences, with limited quantitative data on health outcomes or the aforementioned cost-effectiveness.

## Conclusion

The Refugee Health ICT program demonstrates the potential of integrated, multidisciplinary approaches to support refugee health and primary care access. While implementation challenges existed, the model showed promise in facilitating care transitions and providing comprehensive support to both patients and providers. Key successes include exceeding transition targets, providing valuable wraparound services, and fostering cross-sector collaboration. Future research should focus on long-term outcomes, cost-effectiveness, and scalability of such models. As health care systems worldwide grapple with providing equitable care to refugee populations [3], the lessons learned from this case study can inform the development and implementation of similar integrated care models. The ICT model’s emphasis on personalized support and system navigation aligns well with the complex needs of refugee populations settling in high-income countries [9]. By bridging the gap between specialized refugee health clinics and the broader primary care system, such programs have the potential to improve health outcomes, enhance system efficiency, and promote the long-term integration of refugees into their new communities. The Refugee Health ICT program offers valuable insights into how integrated, patient-centered care models can be developed and implemented to address the unique challenges faced by refugees in accessing and navigating health care systems in their new homes.

## Data Availability

The datasets generated or analyzed during this study are not and will not be made publicly available, as they contain information that could compromise the privacy of research participants, including refugee and asylum claimants.
